# Supplementary material for: Permeation of Light Gases through Hexagonal Ice
Source: Materials (Basel). 2012 Sep 5;5(9):1593–601. doi: 10.3390/ma5091593 (PMC5449016; doi:10.3390/ma5091593)
Supplement: Supplementary File 1 [file materials-05-01593-s001.pdf]

Supplementary Information

1. Crystal Structure Determination

A glass capillary (outside diameter 0.1 mm) was filled with ultrapure water (water column high approximately 5 mm) and allowed to cool at a temperature rate of 10 K/hr down to 260 K followed by 360 K/hr until 150 K. Diffraction data was collected at 150K and then at 195 K and 240 K.

Diffraction data were collected with a Gemini PX Ultra equipped with CuK_{α} radiation (λ = 1.54184 Å), a 4-circle kappa goniometer and a CCD Detector. Data collection and data processing was carried out using CrysAlisPro software from Oxford diffraction. The structures were solved by direct methods using SHELXS-97 [Sheldrick,G.M. SHELXS-97, Program for the solution of crystal structures; University of Göttingen, Germany 1997] with atomic positions and displacement parameters refined with SHELXL-97 [Sheldrick,G.M. SHELXL-97, Program for the refinement of crystal structures; University of Göttingen, Germany 1997]. The oxygen atoms were refined anisotropically.

Precession photographs of the ice crystals were taken to determine the orientation of the *c*-crystallographic axis.

Table S1. Crystal data for ice I_h at 150K, 195K and 240K.

**Crystal data**	**150 K**	**195 K**	**240 K**
Crystal system	Hexagonal	Hexagonal	Hexagonal
Space group	$P6_3/mmc$	$P6_3/mmc$	$P6_3/mmc$
a, Å	4.5167(15)	4.5000(15)	4.492(4)
c, Å	7.290(3)	7.317(4)	7.335(7)
V, Å^3	128.79(8)	128.32(10)	128.16(19)
$R[F^2 > 2\sigma(F^2)]$	0.0614	0.1081	0.0685
$wR[F^2 > 2\sigma(F^2)]$	0.1802	0.2173	0.1725
$\Delta\rho_{max}$ (e Å^{-3})	0.215	0.729	0.272
$\Delta\rho_{min}$ (e Å^{-3})	−0.263	−0.331	−0.384

2. Ice Crystal Porosity

We analyzed the void volume content of crystal ice structures using the software Mercury 2.2 with 0.1 Å of grid spacing [1]

The biggest spherical probe that theoretically could pass through (continuous tubular void volume) had a radius of 1.0 Å (Figure S1). The tubular void volume had the *c*-crystallographic direction. Accordingly, the ice crystals should be considered nonporous since the smallest molecule (He) has a kinetic diameter of 2.6 Å. However, we observed that considerably bigger molecules than He could pass through pointing to the importance of the ice matrix flexibility.

Figure S1. Ice crystal structure at 150 K along the *c*-axis. The void volume accessible to a spherical probe with a radius of 1.0 Å is shown.

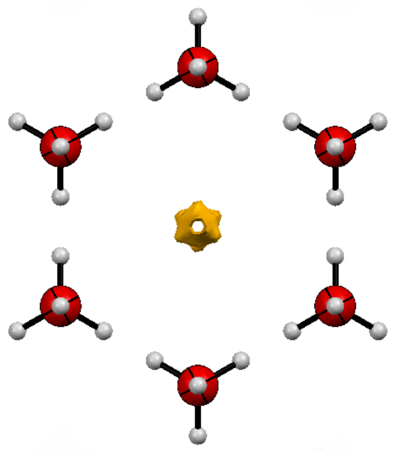

3. Single-Crystal Permeation Experiments

Single-crystal permeation experiments were performed against the atmosphere using a pressurized feed gas chamber as shown in Figure S2 (chamber volume 1.27 mL). Experiments for each gas compounds were always replicated with helium to determine the selectivity with the same ice crystal. Additionally, we noticed that typically disordered ice crystals were more frequently formed at higher temperatures.

The feed pressure could be raised up to at least 7.6 bar without a perceptible disruption of the ice structure (Figure 2).

Figure S2. Scheme of the experimental setup used for the single-crystal permeation experiments.

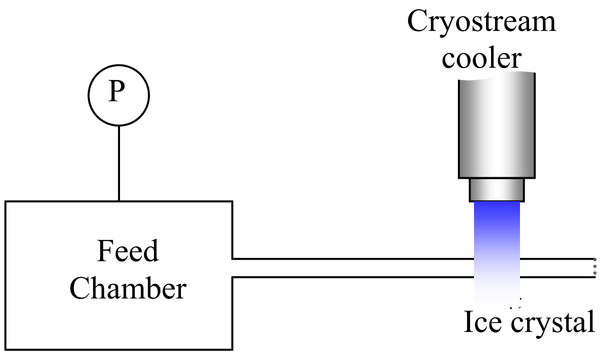

References

1. Macrae, C.F.; Bruno, I.J.; Chisholm, J.A.; Edgington, P.R.; McCabe, P.; Pidcock, E.; Rodriguez-Monge, L.; Taylor, R.; van de Streek, J.; Wood, P.A. Mercury CSD 2.0—New features for the visualization and investigation of crystal structures. *J. Appl. Crystallogr*. **2008**, *41*, 466–470.
